# The Effects of Climate Change on Harp Seals (*Pagophilus groenlandicus*)

**DOI:** 10.1371/journal.pone.0029158

**Published:** 2012-01-04

**Authors:** David W. Johnston, Matthew T. Bowers, Ari S. Friedlaender, David M. Lavigne

**Affiliations:** 1 Duke University Marine Laboratory, Division of Marine Science and Conservation, Nicholas School of the Environment, Duke University, Beaufort, North Carolina, United States of America; 2 International Fund for Animal Welfare, Guelph, Ontario, Canada; University of British Columbia, Canada

## Abstract

Harp seals (*Pagophilus groenlandicus*) have evolved life history strategies to exploit seasonal sea ice as a breeding platform. As such, individuals are prepared to deal with fluctuations in the quantity and quality of ice in their breeding areas. It remains unclear, however, how shifts in climate may affect seal populations. The present study assesses the effects of climate change on harp seals through three linked analyses. First, we tested the effects of short-term climate variability on young-of-the year harp seal mortality using a linear regression of sea ice cover in the Gulf of St. Lawrence against stranding rates of dead harp seals in the region during 1992 to 2010. A similar regression of stranding rates and North Atlantic Oscillation (NAO) index values was also conducted. These analyses revealed negative correlations between both ice cover and NAO conditions and seal mortality, indicating that lighter ice cover and lower NAO values result in higher mortality. A retrospective cross-correlation analysis of NAO conditions and sea ice cover from 1978 to 2011 revealed that NAO-related changes in sea ice may have contributed to the depletion of seals on the east coast of Canada during 1950 to 1972, and to their recovery during 1973 to 2000. This historical retrospective also reveals opposite links between neonatal mortality in harp seals in the Northeast Atlantic and NAO phase. Finally, an assessment of the long-term trends in sea ice cover in the breeding regions of harp seals across the entire North Atlantic during 1979 through 2011 using multiple linear regression models and mixed effects linear regression models revealed that sea ice cover in all harp seal breeding regions has been declining by as much as 6 percent per decade over the time series of available satellite data.

## Introduction

We are currently witnessing significant changes in high latitude ecosystems, manifested most noticeably by rapid declines in the extent of summer ice, significant reductions in perennial ice cover, and declines in sea ice thickness in Arctic and Antarctic regions [1]. Some predictions indicate that annual circumpolar sea ice cover in the Arctic may decline by 20% by 2050 [2], with the possibility of ice-free summers by 2037 [3].

Rapid changes in temperature and ice conditions in Arctic ecosystems pose significant challenges for marine mammals that use sea ice as a platform for breeding and social activity (for reviews, see [4], [5] and [6]). Research conducted to date has tended to focus on waning summer sea ice and the effects on Arctic marine mammals resulting from changes in marine productivity or habitat availability. Less attention has been paid to how changes in seasonal sea ice in adjacent sub-Arctic regions are changing over time. Perhaps most importantly, few studies actually assess the extent to which ice conditions are changing at the spatial and temporal scales relevant to sub-Arctic pinnipeds that rely on seasonal ice during the critical breeding period.

Harp seals (*Pagophilus groenlandicus*) rely on seasonal sea ice as a substrate for pupping and nursing their young [7]. Harp seals have evolved to use transient sea ice as a breeding substrate and have adopted specific life history traits to succeed in this ephemeral environment, including a truncated nursing period (ca. 12 days) after which mothers wean their pups [7]. Despite this adaptation, significant changes in the quality and quantity of ice habitat, and the timing of ice availability for breeding seals may have serious consequences for their populations.

During winters, the North Atlantic Oscillation (NAO) is the dominant pattern in climate variability across the North Atlantic, shaping environmental conditions from Canada to Russia [8]. The NAO index is calculated as the difference in atmospheric pressure between a subtropical high and a polar low [8]. Recent analyses of sea ice dynamics in the breeding habitats of harp seals have revealed that the NAO can greatly effect the quantity and quality of ice available to seals [9] and how persistent these habitats are during spring breakup [10], but no studies have explicitly linked changes in seasonal sea ice driven by NAO variability with effects on seal mortality. In years with reduced ice cover and rapid ice melting, harp seals reportedly suffer significant neonatal mortality [4], [11].

**Figure 1:**
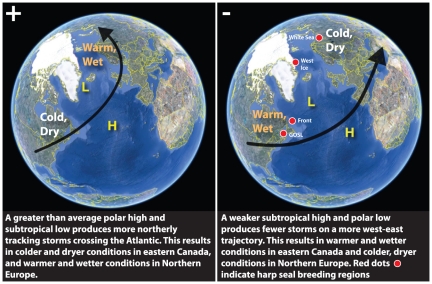
The breeding regions of harp seals (*Pagophilus groenlandicus*) and patterns of the North Atlantic Oscillation (NAO). Red dots illustrate the general breeding locations of harp seals and the effects of both positive (+) and negative (−) phases of the winter NAO on these regions are indicated.

The purpose of the present study was threefold. First, we tested the hypothesis that light ice years in the Northwest Atlantic result in increased neonatal mortality of seals by regressing the number of recorded strandings of dead harp seal pups in the Northeastern United States [12] – an index of natural mortality – against ice cover values from the Gulf of St. Lawrence (the most proximate breeding region) and NAO index values. Building on this relationship, and the established links between ice cover and NAO conditions, we then conducted a retrospective cross-correlation analysis of sea ice and NAO conditions in two breeding regions of harp seals (Figure 1) to assess how changes in ice cover relate to historical observations of neonatal mortality and observed population trends. Finally, we tested the hypothesis that longer-term climate change is affecting the amount of sea ice in the breeding regions of harp seals using linear multiple regression models and linear mixed effects regression models that account for shorter-term variation in ice cover driven by the NAO.

This series of analyses allows us to 1) establish the links between ice cover, NAO conditions and patterns in first year seal mortality over time, 2) provide a novel perspective on previously observed changes in harp seal populations in relation to climate variability and 3) characterize the longer-term warming signal present in sea ice variability in the breeding regions of harp seals across the North Atlantic.

## Methods

### Study Region and Time Frame

To illustrate the recent links between sea ice cover and seal mortality, we used stranding data for dead harp seal pups extracted from the US Northeastern Region stranding dataset covering marine mammal strandings in the Northeastern US from 1993 to 2010 [12], [13]. We then compared these values with sea ice cover data from the Gulf of St. Lawrence during the same time period using linear regression techniques.

For the retrospective analysis of NAO conditions and sea ice, we considered patterns in sea ice cover in two breeding regions of harp seals – the Gulf of St. Lawrence in eastern Canada, and the White Sea region between Norway and Russia – in relation to historical variability in the NAO (Figure 1). These two locations represent the opposite ends of relationships between NAO and sea ice conditions established previously with a shorter time series [9]. We then compared these relationships within a retrospective assessment of published harp seal neonatal mortality data in these regions during 1940 to 2011 [11], [14].

We then conducted a wider examination of sea ice cover across all four breeding locations of harp seals (the above mentioned locations plus the Front off Newfoundland and the West Ice region in the Greenland Sea – see Figure 1) during 1978 to 2011 to assess longer-term trends in sea ice cover across the entire North Atlantic.

### Satellite-derived measures of sea ice cover

We employed sea ice coverage data produced by the US National Sea Ice Data Centre (NSIDC) in Boulder, Colorado, for 1979 to 2011 during February and March, the breeding season of harp seals. These values were derived from NASA Nimbus-7 Scanning Multi-channel Microwave Radiometer (SSMR) data (1979–1987), Defense Meteorological Satellite Program (DMSP) Special Sensor Microwave/Imager (SSM/I) data (satellites F8, F11, and F13 for 1987–2007 – see [15]) and DMSP F17 SSM/I data for 2009 through 2011 [16]. We used mean monthly ice coverages for both breeding sites for all years up to 2007, when the DMSP F13 satellite was lost, after which we used daily ice coverages and averaged the grid values to produce monthly means of sea ice concentration (%) in spatially-explicit harp seal breeding regions defined previously in [9].

### North Atlantic Oscillation Indices

To assess historical changes in climate and sea ice conditions we used winter (December through March) NAO index values (Climate Analysis Section, NCAR, Boulder, USA, Hurrell 1995). These data are based on the difference of normalized sea level pressure between Lisbon, Portugal and Stykkisholmur/Reykjavik, Iceland since 1864.

### Sea ice cover and seal mortality

The effects of changing ice conditions on seal neonatal mortality rates have not been investigated consistently over time, although previous studies indicate that when ice conditions are heavy mortality is generally low (e.g. 1.1–1.4%) [17], and when ice conditions are light it is much higher [11]. To establish current links between changing ice conditions and seal neonatal mortality, we conducted a linear regression of satellite-derived values of sea ice concentration in the Gulf of St. Lawrence during February against yearly stranding rates of dead young-of-the-year (YOY) harp seals in the Northeastern United States (JMP 8.0, SAS Institute) - as an index of natural mortality rates. Specifically, we used stranding network data for the northeastern US Atlantic coast (Maine to Rhode Island) from the Northeast Regional Office of NOAA's National Marine Fisheries Service Marine Mammal Stranding Network during 1993 to 2010 [12], [13]. Since age class was not identified for many of the stranded animals, we calculated the distribution of standard length for identified YOY seals. We then tallied the number of stranded animals in the database whose lengths were less than 1 standard deviation over the mean length of this sample (year 1 animals) and regressed these data against ice cover for that year in the Gulf of St. Lawrence. A similar regression of NAO index values against stranding numbers of dead seals was also conducted to further link seal mortality with climate variability.

### Linking NAO Index Values with Current and Historic Sea Ice Conditions

Satellite-derived values of sea ice concentration were employed to further assess the relationship between winter NAO values and sea ice concentrations identified previously in [10] and [9]. This represents an extension of 5 years to previous time series analyses of ice in the breeding regions of harp seals. We visualized the general trajectory of this time series by fitting a smoothing spline to winter NAO index values using JMP Ver 8.0 (SAS Institute). To confirm previously established linkages between NAO phase and ice conditions, we then conducted a cross-correlation analysis on the NAO and sea ice time series (1979 to 2011) in the Gulf of St. Lawrence and the White Sea breeding regions using JMP 8.0 (SAS Institute). This analysis provides for strong inference on ice conditions in both locations over the extended historical NAO time series.

### Long-term trends of sea ice in harp seal breeding habitats

To investigate the interaction of the NAO with long term trends in ice concentration we initially constructed 3-dimensional wire plots of multiple linear regressions of sea ice concentration against NAO index value, and year for all four breeding regions using the lattice package in R [18]. We statistically assessed ice concentration trends in all four harp seal breeding regions for the months of February and March by fitting a linear mixed effects regression model for each month using the lme4 package in R [19]. These two models included year as a fixed effect and to account for variation caused by the NAO within each region, monthly NAO index values were used as random effects with regions as a grouping factor where:
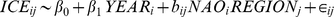
Mixed effects models are advantageous in that they allow modeling and forecasting of non-stationary changes inherent in climate data through the inclusion of predictors as fixed or random effect variables [20].

## Results

**Figure 2:**
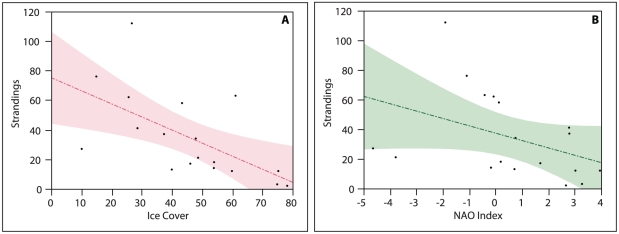
The relationships between harp seal neonatal mortality, the North Atlantic Oscillation (NAO) and sea ice cover. Panel A represents a linear regression of February sea ice cover in the Gulf of St. Lawrence against stranding rates of dead young-of-the-year harp seals in the Northeastern United States. Panel B represents a linear regression of February sea ice cover in the Gulf of St. Lawrence and winter North Atlantic Oscillation (NAO) index values.

A total of 365 dead harp seals specifically identified and reported as YOY animals stranded in the Northeastern US during 1993 to 2010. The distribution of standard lengths was normal (Shapiro-Wilk test, p>0.05) and the mean length of YOY animals was 105 cm with a SD of 9.5 cm. Using the upper range of lengths (115 cm) and avoiding animals listed specifically as sub-adults regardless of standard length, we then identified a total of 693 YOY seals in the stranding database and extracted these records for linear regression modeling. The linear regression of seal strandings against ice cover in the Gulf of St. Lawrence (the most proximate breeding location) is presented in Figure 2 (A). The regression model revealed a strong negative correlation between ice cover and stranding rates (n = 18, p = 0.008, r^2^ = 0.37), where lighter ice conditions correlate with increased numbers of stranded dead seals. The regression of NAO index values and seal mortality (Figure 2 B) revealed a similar but less robust relationship (n = 18, p = 0.09, r^2^ = 0.16). See Table 1 for details on both ice cover and NAO regressions.

**Table 1:** Term estimates and standard errors for linear regressions of yearly stranding numbers of dead young-of-the-year harp seals in the Northeastern United States and (A) February sea ice cover in the Gulf of St. Lawrence breeding region and (B) winter North Atlantic Oscillation (NAO) index values.

Model	Term	Estimate	Std Error
(A) Strandings by Ice Cover	Intercept	75.22	14.69
	Ice Cover	−0.88	0.29
(B) Strandings by NAO Index	Intercept	37.31	6.70
	NAO Index	−4.97	2.80

**Figure 3:**
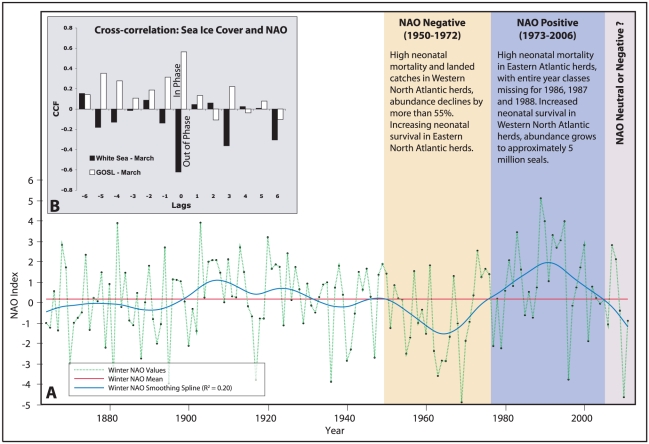
Retrospective analysis of winter North Atlantic Oscillation (NAO) index during 1865 to 2011 and sea ice conditions. Panel A illustrates the time series fit with a smoothing spline to illustrate the general pattern of the climatic signal. The inset (B) illustrates the relationships between sea ice cover and NAO conditions in the Eastern North Atlantic (out of phase) and Western North Atlantic (in phase). Observed effects of NAO phase on neonatal mortality of harp seals (*Pagophilus groenlandicus*) indicated for extended negative and positive NAO phases.

The relationships between sea ice conditions and NAO index in the White Sea and Gulf of St. Lawrence breeding regions during March 1979 to 2011 are presented in Figure 3 (A). As predicted, eastern North Atlantic sea ice cover and NAO conditions were out of phase – with heavier ice cover during negative NAO periods and lighter ice cover during positive NAO periods. In contrast, western North Atlantic ice conditions were in phase with NAO conditions – with heavier ice cover during positive NAO conditions and lighter ice cover during negative NAO periods.

The general trajectory of the winter NAO index from 1865 to 2011 is illustrated in Figure 3 (B). During 1949 to 1973, the winter NAO index was consistently negative. During this period ice conditions were heavy in the western North Atlantic, and light in the Northeast Atlantic. Following this, there was an extended positive period of winter NAO averages during 1974 to 2004. Ice conditions in the Northeast Atlantic were light during this period, and heavy ice conditions prevailed during this time in the Northwest Atlantic. These general relationships have been partially described previously [9], [10]. Subsequent to 2004, it appears that neutral to negative winter NAO indices have become dominant (Figure 3 B).

**Figure 4:**
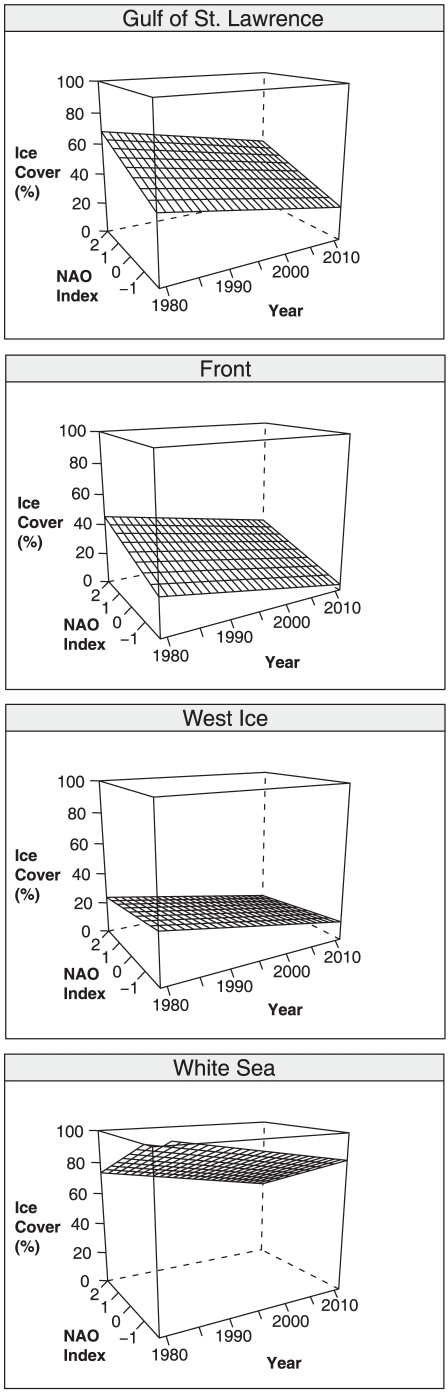
Changes in sea ice cover in harp seal breeding regions. These wireframe plots illustrate satellite-derived measures of sea ice cover during the month of February at all four breeding regions of harp seals (*Pagophilus groenlandicus*) in relation to winter North Atlantic Oscillation (NAO) index values during 1979 to 2011.

A series of three-dimensional wire plots that display the linear relationships between sea ice cover in harp seal breeding regions during the month of February as a function of NAO index over our entire time series (1979 to 2011) are presented in Figure 4. The results for March showed similar but less extreme declines of sea ice concentration over time. Both multiple regression models (February and March) were significant (p<0.05), and year was a significant predictor for sea ice concentration in February (p<0.05) and approached significance for March (p = 0.08). The multiple r-squared values for these regressions were low (0.12 and 0.09, for February and March respectively) due to variation in NAO effects on ice cover. These plots are useful, however, as they graphically illustrate the general relationships between sea ice cover and NAO conditions revealed by our mixed effects regression models.

The results of our mixed effects models reveal a statistically significant annual decline of sea ice cover in all four breeding regions during the study period, regardless of variation in NAO conditions. Both monthly model coefficients were significant at the 95% percent confidence interval. February ice concentration declined at a rate of 0.63±0.12% per year from 1979 to 2011 (table 1). The model for March revealed a smaller annual decline of 0.41±0.13% over the same time span (Table 2). Group level effects fell in line with expected values for each region (Table 3), although the influence of the NAO on ice in the West Ice region was negligible.

**Table 2:** Fixed effects for mixed-effects regression model of sea ice cover in the Gulf of St. Lawrence (GOSL), the Front, the West Ice and White Sea breeding regions of harp seals (*Pagophilus groenlandicus*.

Fixed Effects	Estimate	Std. Error
**February**		
(Intercept)	52.65	11.44
I(Year - 1979)	−0.63	0.12
**March**		
(Intercept)	47.04	9.36
I(Year - 1979)	−0.41	0.13

**Table 3:** Group effects for mixed-effects model of sea ice cover in the Gulf of St. Lawrence (GOSL), the Front, the West Ice and White Sea breeding regions of harp seals (*Pagophilus groenlandicus*).

Group Effects	(Intercept)	NAO Index
**February**		
GOSL	2.65	4.51
Front	−17.96	4.51
West Ice	−24.95	−0.99
White Sea	35.28	−6.59
**March**		
GOSL	−2.30	6.83
Front	−17.50	7.14
West Ice	23.59	0.20
White Sea	33.52	−9.67

## Discussion

### Short-term Climate Variability

The linear regressions of ice cover in the Gulf of St. Lawrence and NAO index values against stranding rates of YOY seals in the Northeastern US provide a useful proxy for changes in seal mortality associated with reductions in ice cover driven by NAO variability in the Northwestern Atlantic. Strandings data from the same region have been used previously to assess spatial patterns in stranding rates of ice seals [12], as well as to assess patterns in neonatal mortality rates of right whales (*Eubaleana glacialis*) in the same region [21]. Indeed, large scale assessments of stranding data in relation to visual survey data from several countries indicate that assessments of stranding rates often outperform visual surveys in determining local diversity, and they are extremely useful for studying spatial and temporal ecology of marine mammal populations, especially for time series greater than 10 years [13]. Our regressions reveal that an increase in first year seal mortality occurs in years with lighter ice cover and lower NAO index values, consistent with earlier qualitative observations reported in the literature [10], [11], [12].

North Atlantic Oscillation conditions greatly affect sea ice dynamics in harp seal breeding regions. When the winter NAO is negative, sea ice cover tends to be below average in the breeding locations of harp seals on the east coast of Canada and this has contributed to significant mortality of neonatal harp seals through a combination of interrupted nursing, starvation, cold stress and crushing by shifting floes when prematurely forced into the water by the rapid melting and break-up of ice [11].

While natural mortality rates for this population (or indeed for any other population of ice seals) have not been produced for the range of ice/NAO conditions captured by our times series, our linear regression of ice cover and stranded dead harp seals in the Northeastern US provides further evidence that young harp seals fare poorly in light ice years.

This is not a recent phenomenon. For example, harp seals were forced to prematurely vacate whelping patches in the Gulf of St. Lawrence during the winter of 1966 [11], a strongly negative NAO year. Ice records indicate that 1966 was a light ice year in eastern Canada [11] and the station-based NAO index value for this year was −1.86. In 1969 the winter NAO value was also strongly negative (−4.89) and sea ice cover in the Gulf of St. Lawrence was the lowest on record [10]. During this year thousands of seals were crushed in moving ice, or were prematurely forced to vacate whelping patches during rapid ice breakup [11]. In 1998 and 1999, both years with low winter NAO indices and light ice conditions, thousands of dead seals washed ashore on the beaches of Cape Breton [22]. Light ice conditions have been observed during 11 of the past 14 years (1996–2011) in the Gulf of St. Lawrence, and many of these years exhibit significant negative winter NAO anomalies and in many cases high seal mortality [23]. Indeed, in recent years (1996 onwards) the winter NAO has exhibited a greater number of neutral and negative periods (Figure 2 A), indicating that a switch to a more consistently negative phase - as seen in the 1950s through the 1970s - may be occurring.

Harp seals numbers now appear to be plateauing in the Northwest Atlantic off eastern Canada [24], concomitant with a downward trend in NAO indices and increasingly light ice conditions during 1996 to 2010. In recent years neonatal mortality has been extremely high, and potentially resulted in the loss of entire year classes in the southern Gulf of St. Lawrence [4], [23]. The second latest year in our time series (2010) is no exception to this link between NAO index, ice conditions and neonatal mortality. In that year, the winter NAO index value dropped to −4.64, ice conditions were the lightest in the satellite record in the Gulf of St. Lawrence, and harp seal neonatal mortality reportedly approached 100% in this region [4].

### A Novel Perspective on Fluctuating Harp Seal Populations

There have been dramatic changes in the trajectory of harp seal populations across the North Atlantic over time, and these fluctuations have never been fully explained. Several hypotheses have been offered to explain fluctuations in harp seal abundance across the Atlantic during our study period, including overexploitation, by-catches and changes in prey availability [14], [25]. However, none have addressed climate-related changes in breeding habitat as a significant source of mortality.

A large reduction in harp seal abundance in the Northwest Atlantic occurred during the 1950s through the early 1970s, with estimates ranging between 50 and 66% declines in seal numbers [11], [26]. Our retrospective assessment reveals that during this period the NAO was consistently negative (Figure 2 A). As sea ice conditions in the Northwest Atlantic are in phase with NAO conditions (Figure 1), this period of time would have exhibited consistently light ice conditions. Our retrospective analysis also reveals that 1973 to 2000 was period of consistently positive NAO conditions (Figure 2 A), during which sea ice conditions in the Northwest Atlantic would have been heavy and more conducive to successful reproduction in harp seals. During this period the Northwest Atlantic harp seal population grew consistently, with estimates surpassing 5 million animals [24].

Contrary to the above relationship in the Northwest Atlantic, sea ice conditions in the Northeast Atlantic (White Sea) are out of phase with the NAO (Figure 1 A); during positive NAO periods sea ice conditions tend to be light [9]. Harp seals in the Northeast Atlantic declined from approximately 1.5 million seals in the early 1950s to 500,000 in the early 1960s [14]. After this, the population recovered to approximately 800,000 by 1978 [14]. Soon thereafter, survival of neonatal harp seals in the Northeast Atlantic declined, and remained low until the early 1990s. In particular, neonatal mortality during 1986, 1987 and 1988 was extremely high, resulting in an almost complete absence of these year classes when sampled in the 1990s [14]. The period of increasing neonatal recruitment and abundance in Northeast Atlantic harp seals (1960 to 1973) corresponds with the extended negative NAO regime (Figure 2 A) during which sea ice cover was at or above average conditions in the Barents/White Sea region [9]. The period of decreasing recruitment in these animals (1982 to 1992) corresponds with the extended positive NAO regime (Figure 2 A) during which sea ice conditions were in decline or light [9].

Hunting mortality may also affect the population dynamics of harp seals across the Atlantic, especially when viewed as a cumulative stressor on reproductive output of a population. Harp seals are hunted commercially and for subsistence purposes across their range. In the Northwest Atlantic, total landed catches of harp seals during the extended negative NAO regime (1950 to 1972) were relatively high – ranging up to 389,410 seals annually. This hunt focused on newborn whitecoat pups [7]. Landed catches were smaller during the first part of the extended positive NAO regime (1973 to 1982), ranging up to 202,169 seals. However, from 1983 to 1995, catches dropped further to a maximum 94,046 seals per year and the focus of the hunt shifted from whitecoats to older, moulted pups (beaters, aged about 3 weeks to 3 months). Catches increased again during 1996 to 2005, ranging up to 365,971 seals [27]. It should be noted that in some regions the magnitude of hunting mortality has been lower than the level of natural mortality reported in poor ice years, when virtually all pups born (e.g. southern Gulf of St. Lawrence) have reportedly died [4].

The results of our retrospective analysis reveal that harp seal populations across the Atlantic appear to have fluctuated in synchrony with NAO trends and associated ice conditions. As such, we hypothesize that changing ice conditions may have contributed to the population dynamics of harp seals across the North Atlantic, most likely through periods of sustained reproductive failure or success, coupled with hunting and other environmental factors. Further research is required to assess how combinations of removals by sealers and recent (1996 to 2011) ice-induced increases in neonatal mortality will affect current and future harp seal populations.

### Long-term Sea Ice Trends in harp seal breeding locations

Our mixed-effects model effectively extracts the long-term warming signal from our time series of sea ice cover values that is dominated by NAO variability. Our model indicates that sea ice in all harp seal breeding regions is in decline regardless of the influence of the NAO, at a rate of approximately 6% per decade in February, and 4% per decade in March (this lower rate of decline likely stems from the fact that the majority of sea ice loss happens in February). This overall decline is less dramatic than recently observed conditions in eastern Canada (2000 to 2010), and it appears that the large scale effects of recent warming at high latitudes may have been obscured in the Northwest Atlantic in part by the effects of a consistently positive winter NAO regime on seasonal sea ice development and persistence during 1980 to 2000.

Our results are the first to illustrate that ice cover in the breeding habitats for harp seals across the North Atlantic has been in decline since the beginning of the satellite record of ice conditions (1979). Along with observed increases in YOY mortality in the Northwest Atlantic harp seal herds indicated above, the consistent decline in seasonal sea ice cover across the North Atlantic has also resulted in a recent sharp reduction in pup production in the White Sea harp seal population [28].

Recent records of harp seals whelping off East Greenland (the West Ice) indicate that some response of the animals to phenological shifts in breeding habitat due to warming may be occurring [4], [29]. In the Northwest Atlantic, however, it appears that many seals have been returning to traditional breeding grounds in the Gulf of St. Lawrence and on the Front, off Newfoundland, regardless of ice conditions. This observation may reflect a lack of plasticity in their breeding system, which has likely evolved in response to a combination of factors including photoperiod [30], predation pressure [7], [31] and the long-term predictability of sea ice in traditional breeding regions. Further research is required to assess the timing of ice breakup and parturition patterns to determine how harp seals are responding to rapid warming in their breeding regions. It should also be noted that fitted values from mixed effects regression models are generally considered conservative estimates. This is referred to as the “shrinkage effect” [32]. In light of this, our model may underestimate total ice decline in the breeding regions of harp seals over the time of our study.

Harp seals have evolved to use seasonal sea ice as a breeding substrate and have adopted specific life history traits to succeed in this environment. Considering this, they are well suited to deal with natural shifts in climate, including the effects of the NAO on sea ice conditions. However, these animals may not be well adapted to absorb the cumulative effects of human influences (primarily hunting and to a lesser extent by-catch – which can be high in some years [33], [34]), short-term climate variability and global warming. Other ice-associated seals are also likely to be vulnerable to these combined effects, and share many of the breeding regions and life history traits of harp seals. In particular, hooded seals (*Cystophora cristata*) may be especially at risk. The Northwest Atlantic stock appears to be stable at present, but the Northeast Atlantic stock, which breeds on the West Ice, off the east coast of Greenland, has declined by 85–90% over the last 40–60 years, prompting a listing of this species as *Vulnerable* on the IUCN Red List of Threatened Species [35].
